# Contextual patterns in publicly reported non-traumatic sudden deaths among university students in physical education and campus sports settings in China: a retrospective thematic analysis

**DOI:** 10.1186/s12889-026-28079-5

**Published:** 2026-06-08

**Authors:** Yuanyuan Li

**Affiliations:** School of Physical Education, Sichuan Minzu College, Kangding, Sichuan China

**Keywords:** University physical education, Non-traumatic sudden death, Public case reports, Thematic analysis, Campus sports safety, Emergency preparedness, Health governance

## Abstract

**Background:**

Physical education classes, physical fitness testing, and other campus-based sports activities are important settings in which university students engage in organized and semi-organized physical activity. Although non-traumatic sudden death in these settings is rare, its consequences are severe for students, families, universities, and campus public health governance. Existing research has mainly focused on pathological mechanisms, clinical interpretation, emergency medicine, and legal responsibility, whereas less attention has been paid to how such events are publicly represented and what recurrent contextual patterns are visible in university sports settings. This study aimed to identify recurrent public-record-based contextual patterns reported in cases of non-traumatic sudden death among university students in physical education and campus sports-related settings in China, and to discuss their implications for campus sports safety governance and public health prevention.

**Methods:**

A retrospective qualitative thematic analysis was conducted using publicly available Chinese-language case materials. Public reports, university notices, education authority notices, institutional statements, and news reports were searched for cases occurring between 1 January 2016 and 25 December 2025 in mainland China. Cases were included if they involved an enrolled university student, occurred during a physical education class, physical fitness test, campus competition, training session, extracurricular campus sport, or another clearly identifiable campus sports-related setting, and were publicly described as non-traumatic sudden death, cardiac arrest followed by death, respiratory and cardiac arrest followed by death, or acute collapse during or shortly after exercise followed by unsuccessful rescue. Multiple reports relating to the same incident were merged into case files. Source reliability and completeness were assessed according to source authority, proximity to the event, information completeness, cross-source consistency, and transparency of event description. Reflexive thematic analysis was then conducted. Five trained coders assisted with preliminary coding, while the author integrated coding outputs, developed final categories and themes, and interpreted the findings.

**Results:**

Twenty-one publicly reported fatal cases were included in the main analytical sample. Six recurrent public-record-based contextual themes were identified: individual health vulnerability; mismatch between task demands and physical preparedness; limited visibility of health screening and classroom monitoring; incomplete public reporting of emergency preparedness and on-site rescue capacity; evaluative contexts and possible barriers to symptom reporting; and the amplifying role of environmental conditions. These themes were interpreted as contextual signals visible in public reports rather than as clinically verified causal risk factors.

**Conclusions:**

Publicly reported non-traumatic sudden deaths among university students in physical education and campus sports-related settings appear to be publicly represented within multiple interacting individual, task-related, institutional, psychosocial, environmental, and emergency-response contexts. The findings should not be interpreted as evidence of causal risk factors, incidence, population-level association, or confirmed institutional responsibility. Rather, they provide a public-record-based synthesis of recurrent contextual patterns that may inform hypothesis generation, campus safety communication, health screening, task stratification, symptom-reporting mechanisms, emergency preparedness, and future empirical research on campus sports safety governance.

**Supplementary Information:**

The online version contains supplementary material available at https://doi.org/10.1186/s12889-026-28079-5.

## Background

Physical education classes, physical fitness testing, and other campus-based sports activities are important components of university health promotion and student development. In higher education, these activities contribute not only to physical fitness, motor competence, and lifelong exercise habits, but also to mental health, social participation, and the cultivation of healthy behaviors. At the same time, university physical education and campus sports settings are institutionalized environments in which exercise participation is organized, monitored, evaluated, and supported by universities. Therefore, sports-related safety incidents in these settings should be understood not only as individual medical events, but also as issues of campus public health governance, campus safety communication, and emergency preparedness.

Non-traumatic sudden death during or shortly after exercise is rare among young people, but its consequences are severe for students, families, teachers, universities, and wider society. In university contexts, such events often attract public attention because they occur in educational settings that are expected to promote health and safeguard students. Existing research on exercise-related sudden cardiac arrest and sudden cardiac death has mainly focused on pathological mechanisms, cardiovascular screening, emergency response, and survival outcomes. These studies have provided important evidence regarding underlying cardiovascular abnormalities, exertional triggers, cardiopulmonary resuscitation, automated external defibrillator use, and emergency action planning [1–5]. However, less attention has been paid to how fatal incidents in university physical education and campus sports settings are publicly represented, what contextual information is visible in public reports, and what recurrent patterns may be identified across publicly reported cases.

In China, public reports of non-traumatic sudden death among university students in physical education classes, physical fitness tests, campus competitions, and extracurricular sports activities have appeared intermittently over the past decade. These reports often mention exercise tasks such as running, physical fitness testing, basketball, sports-meet events, or pre-competition training. Some cases also refer to possible underlying disease, sudden collapse, emergency rescue, hospital transfer, or institutional follow-up. Nevertheless, the publicly available information is often incomplete and uneven. Medical records, autopsy findings, full emergency timelines, pre-exercise screening records, and internal institutional investigation files are usually unavailable to researchers. As a result, such public materials cannot support definitive medical causality, incidence estimation, or robust statistical association.

This limitation does not mean that publicly reported cases have no research value. Rather, when interpreted cautiously, they can provide a window into the kinds of contextual signals that become visible in the public record. These signals may include the setting in which the event occurred, the type of activity being performed, whether health vulnerability was reported, how screening and monitoring were described, whether students were in evaluative or competitive contexts, what emergency response was publicly reported, and what institutional follow-up was made visible. A public-document-based qualitative analysis can therefore contribute to hypothesis generation and campus safety reflection, provided that its methodological boundaries are explicitly stated.

The present study is informed by a socio-ecological and systems-oriented perspective on campus health and safety. From this perspective, exercise-related fatal events should not be interpreted solely through individual pathology or a single exercise task. They are represented within layered contexts involving individual health vulnerability, exercise-task demands, institutional screening and monitoring, psychosocial pressure, environmental exposure, and emergency preparedness. This perspective is particularly relevant to university physical education, where participation is shaped by curriculum requirements, fitness-testing systems, teacher monitoring, peer norms, institutional risk-management procedures, and the availability of emergency resources.

However, existing discussions of student sudden death in campus sports settings often remain fragmented. Some studies and public debates emphasize medical conditions or individual responsibility, while others focus on school management, legal accountability, or emergency rescue. Few studies have systematically organized publicly reported cases to examine how multi-level contextual patterns are represented across incidents. Moreover, previous analyses have not always clearly distinguished between clinically verified risk factors and public-report-based contextual patterns. This distinction is important because publicly available materials can show what was reported, but they cannot independently verify the actual cause of death, the completeness of institutional procedures, or the true prevalence of specific risk conditions.

To address this gap, the present study conducted a retrospective thematic analysis of publicly reported cases of non-traumatic sudden death among university students in physical education and campus sports-related settings in China from 2016 to 2025. The study does not aim to determine medical causality, estimate incidence, assign institutional responsibility, or identify statistically verifiable risk factors. Rather, it aims to identify recurrent contextual patterns visible in public reports and to discuss their implications for campus sports safety governance, prevention-oriented communication, health screening, task stratification, and emergency preparedness.

Specifically, this study addresses the following questions: (1) What kinds of public-report-based contextual information are available in cases of non-traumatic sudden death among university students in physical education and campus sports-related settings? (2) What recurrent public-record-based contextual patterns can be identified across these cases? (3) How can these patterns inform prevention-oriented discussion and campus sports safety governance while avoiding causal overinterpretation?

By answering these questions, the study seeks to provide a transparent and cautious synthesis of publicly reported cases. Its contribution lies not in establishing epidemiological risk factors, but in clarifying how recurrent contextual signals visible in the public record may inform institutional reflection and future empirical research on university sports safety.

## Methods

### Research design and theoretical orientation

This study adopted a retrospective qualitative design based on publicly available case materials. The purpose was not to estimate incidence, determine medical causality, or identify statistically verifiable risk factors, but to examine how non-traumatic sudden deaths in university physical education and campus sports-related settings were publicly described and what recurrent contextual patterns could be identified across cases.

The study was informed by a socio-ecological and systems-oriented perspective on campus health and safety. From this perspective, exercise-related fatal events are not interpreted solely as individual medical events, but as incidents occurring within layered contexts that include individual health vulnerability, exercise-task demands, institutional screening and monitoring, help-seeking and reporting norms, environmental exposure, and emergency preparedness. This theoretical orientation guided the extraction and interpretation of case information, while the coding itself remained primarily inductive.

In the analysis, this perspective functioned as a sensitizing framework rather than a causal model. It guided attention to individual, task-related, institutional, psychosocial, environmental, and emergency-response dimensions, while allowing codes and themes to be developed inductively from the public case materials.

Because the study relied on public reports rather than clinical records, institutional investigation files, interviews, or prospective surveillance data, the analysis was deliberately framed as a thematic synthesis of public-record-based contextual patterns. The resulting themes should therefore be understood as analytically informative contextual signals rather than causal risk factors or population-level associations.

### Data sources and search strategy

Publicly accessible online materials were used as the primary data source. These included university notices, education authority notices, official institutional statements, mainstream news reports, local news reports, and other publicly available web-based materials. The search covered incidents occurring between 1 January 2016 and 25 December 2025 involving university students in mainland China.

The case retrieval process was conducted primarily in Chinese because the events of interest occurred in China and were mainly reported in Chinese-language public sources. Searches were conducted through general web search engines and publicly accessible news platforms. The search terms combined words referring to the population, setting, activity, and outcome. The main keyword groups were as follows: (1) population and institution: “大学生”, “高校学生”, “高校”, “大学”, “university student”, and “college student”; (2) physical education and sport setting: “体育课”, “体测”, “体质测试”, “校园跑步”, “篮球”, “羽毛球”, “运动会”, “训练”, “比赛”, and “选拔赛”; (3) event outcome: “猝死”, “心脏骤停”, “呼吸心跳骤停”, “晕倒”, “倒地”, “抢救无效”, and “死亡”; and (4) governance or incident-related terms: “通报”, “情况说明”, “学校回应”, “安全事故”, and “调查”.

Searches were performed using combinations of these terms, for example: “大学生 体育课 猝死”, “高校 体测 猝死”, “大学生 1000米 体测 猝死”, “大学生 800米 猝死”, “大学生 篮球 猝死”, “高校 体育课 抢救无效”, “大学生 运动会 猝死”, and “高校 学生 心脏骤停 死亡”. For each potentially eligible incident, additional targeted searches were conducted using the institution name, date, activity type, and key event description to identify duplicate reports, official statements, follow-up reports, and more detailed accounts.

Because public web searches are dynamic and search-engine dependent, the retrieval process was documented as a case-identification log rather than as a reproducible database search. The final search update was completed on 25 December 2025. Direct web links and source information for the included cases are provided in Additional file 1.

### Operational definition and case eligibility

For the purposes of this study, “non-traumatic sudden death” was operationally defined as a publicly reported fatal event involving sudden collapse, cardiac arrest, respiratory and cardiac arrest, or acute loss of consciousness during or shortly after a campus physical education or sports-related activity, followed by unsuccessful rescue, without publicly reported evidence of trauma, collision injury, equipment injury, drowning, suicide, self-harm, or criminal injury. This operational definition was based on how the events were described in public records and should not be interpreted as an independent clinical diagnosis.

To distinguish exercise-related cases from coincidental deaths occurring on campus, cases were included only when the public record clearly linked the collapse or fatal deterioration to a physical education class, physical fitness test, campus competition, selection activity, training session, extracurricular campus sport, or another identifiable sports-related activity. Cases in which a student died in a dormitory, classroom, or general campus location without a verifiable exercise-related context were excluded or treated as boundary cases.

This study therefore did not classify all sudden deaths among university students as exercise-related. Instead, it focused on cases in which the public record made the physical education or campus sports context sufficiently clear for case-level extraction and thematic coding.

### Case screening and selection process

Potentially relevant records identified during the search were first screened by title, headline, and short description. Records were then assessed at the full-text level when they appeared to involve a university student, a campus physical education or sports-related setting, and a fatal non-traumatic event. Multiple reports referring to the same incident were grouped and treated as one case file. When several reports described the same event, the most authoritative and information-rich source was used as the primary source, while other reports were used for cross-checking and contextual supplementation.

Cases were included if they met all of the following criteria: (1) the event occurred in mainland China between 1 January 2016 and 25 December 2025; (2) the individual was an enrolled university or college student; (3) the event occurred during a physical education class, physical fitness test, campus competition, selection activity, training session, extracurricular campus sport, or another clearly identifiable campus sports-related setting; (4) the event was publicly described as sudden collapse, non-traumatic sudden death, cardiac arrest followed by death, respiratory and cardiac arrest followed by death, or acute collapse during or shortly after exercise followed by unsuccessful rescue; and (5) the available public materials contained sufficient contextual information to support case-level extraction and thematic coding.

Cases were excluded if they involved trauma, collision injury, equipment injury, drowning, suicide, self-harm, criminal events, non-sports-related dormitory or campus settings, or cases in which the exercise-related context could not be verified. Cases were also excluded when available reports were too brief to extract essential information on setting, activity, outcome, and response.

**Fig. 1:**
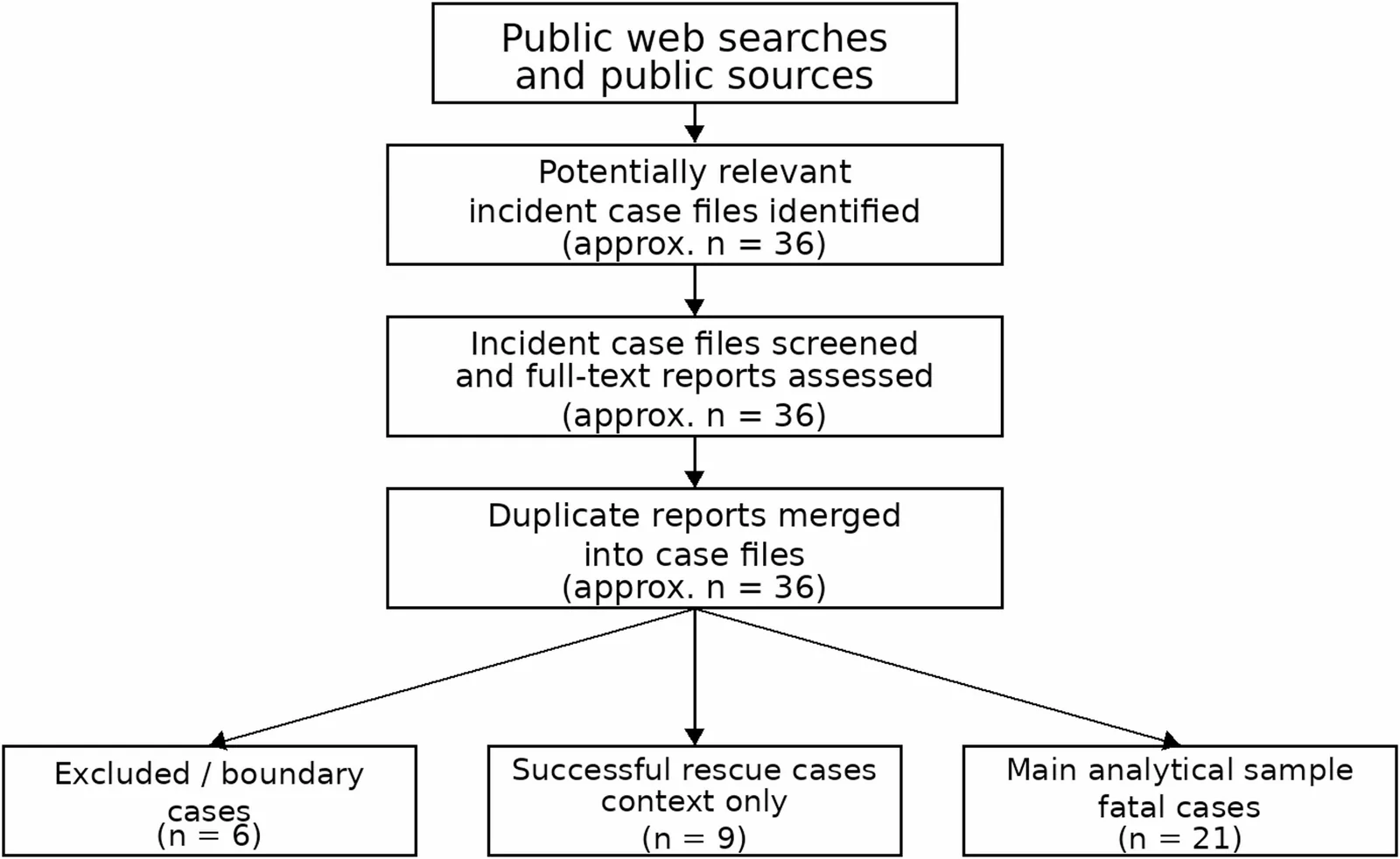
Flow diagram of case identification, screening, deduplication, and inclusion with approximate case-level counts

The screening process is summarized in Fig. 1. Boundary cases that were considered but excluded from the main analytical sample are presented in Table 1 to clarify the scope of the study. Because public web searches are dynamic, duplicate-prone, and partly dependent on search engine indexing, exact record-level retrieval counts could not be reconstructed reliably. Therefore, Fig. 1 reports approximate case-level counts rather than systematic-review-style database counts. The final analytical sample included 21 publicly reported fatal cases. Boundary excluded cases (*n* = 6) and supplementary successful rescue cases (*n* = 9) were retained only for contextual clarification and were not analyzed as part of the fatal-case sample.

**Table 1:** Supplementary boundary cases

Case ID	Date	University	Event Type	Setting	Core Description	Included in Main Sample	Reason for Exclusion
E1	2017-06-16	China Pharmaceutical University	Major safety accident	Swimming class	A student drowned during a swimming class and died despite rescue efforts.	No	This was a major sports teaching-related safety accident rather than sudden death.
E2	2022-11-08	Hebei Institute of Communications	Sudden death in dormitory	Felt unwell in dormitory after exercise	A student felt unwell after exercise upon returning to the dormitory and later died despite rescue efforts.	No	Public reports did not confirm that the death occurred directly during a physical education class, training session, fitness test, or sports activity.
E3	2023-05-06	Hainan Vocational University of Science and Technology	Sudden death on campus	Abnormal condition on campus	A student developed an abnormal condition on campus and died despite rescue efforts. A forensic examination determined that the death was caused by an underlying medical condition.	No	Public information did not confirm that the event occurred during physical education, fitness testing, training, or another clearly identifiable sports setting.
E4	2024-10-19	Guilin University	Sudden death in dormitory	Dormitory	A student suddenly collapsed in the dormitory and later died in hospital. The cause of death was reported as cardiogenic sudden death and acute myocardial infarction.	No	This occurred in a dormitory setting rather than in a sports-related setting.
E5	2025-05-19	Chongqing College of International Business and Economics	Campus facility safety accident	After physical education class	After a physical education class, a student passed near the basketball court and fell into a septic tank in the grass, resulting in death.	No	This was a campus facility safety accident rather than exercise-related sudden death.
E6	2025-09-06	Chongqing College of Mobile Telecommunications	Sudden death in dormitory	Dormitory	A student was found without vital signs in the dormitory. Police ruled out criminal involvement and the death was considered consistent with sudden death.	No	This occurred in a dormitory setting rather than in a sports-related setting.

The events listed in this table were relevant to student safety incidents in higher education but were not included in the main analytical sample of this study. This table clarifies the study boundaries and improves the transparency of case screening and the completeness of the research materials

### Source quality assessment

Because this study relied on publicly available media and institutional materials, a structured source quality assessment was conducted to evaluate the reliability and completeness of each case file. Each source was assessed according to five dimensions: source authority, source proximity to the event, information completeness, cross-source consistency, and transparency of event description.

Source authority was rated higher when information came from university notices, education authority notices, police or official investigation statements, or established mainstream media outlets. Source proximity referred to whether the report was issued close to the time of the event or based on direct institutional response. Information completeness referred to the availability of key details, including date, institution, setting, activity, collapse process, reported outcome, emergency response, and institutional follow-up. Cross-source consistency was assessed by comparing multiple reports of the same event where available. Transparency referred to whether the report clearly distinguished confirmed information from speculation, family statements, media interpretation, or public commentary.

Cases based only on fragmentary or unverifiable information were excluded from the main analytical sample. For included cases, source limitations were retained in the case file and considered during coding. Information that was absent, uncertain, or inconsistent across sources was coded as “not reported” or “unclear” rather than treated as evidence. Public descriptions of cause of death were recorded as reported by the sources and were not interpreted as independent clinical determinations by the author.

To further integrate source quality into the main manuscript, Table 4 summarizes the main source types used for the 21 included fatal cases and their source-quality interpretation. Source type was classified according to the main source basis used for each case file. When multiple reports were available for the same incident, official or institutional sources were prioritized, and other reports were used only for cross-checking or contextual supplementation.

### Data extraction and case file construction

After screening, each eligible event was organized into an individual case file. A structured extraction framework was used to record the year and date of occurrence, institution, province or municipality where available, setting type, sex where reported, sport or activity involved, public description of collapse or symptom onset, public description of cause of death, reported health information, screening or monitoring information, psychosocial or evaluative context where available, on-site emergency response, hospital transfer or rescue outcome, environmental conditions where available, source type, and source limitations.

When several reports described the same case, information was compared across sources and merged into a single case file. If reports differed in detail, priority was given to official university notices, education authority notices, police or investigation statements, and more information-rich mainstream reports. Less authoritative sources were used only to supplement contextual information when they were consistent with higher-priority sources. Unverified claims, online commentary, and speculative interpretations were not used as confirmatory evidence.

The 21 included fatal cases are summarized in Table 2. Supplementary boundary cases excluded from the main analytical sample are presented in Table 1. Supplementary successful rescue cases relevant to emergency preparedness are presented in Table 3. A case-level source inventory with direct web links for the included fatal cases is provided in Additional file 1.

**Table 2:** Main analytical fatal cases, 2016–2025

Case ID	Date	Setting	Sex	University	Sport/Activity	Cause-of-Death Description	Response	Source
1	2016-10-11	Physical education warm-up activity	Male	Changzhou Institute of Light Industry Technology	Preparatory activity before physical education class	Sudden death	On-site first aid, emergency call, hospital transfer; rescue unsuccessful.	Mainstream media source
2	2016-11-04	Physical education / fitness testing	Male	Chongqing University of Posts and Telecommunications	Physical fitness testing during physical education class	Collapse followed by death despite rescue	University statement; hospital rescue unsuccessful; police involved.	Aggregated web source / public report
3	2016-11-05	Campus competition / faculty sports meet	Male	Guangdong University of Technology	800-meter race	Hospital diagnosis: cardiogenic sudden death	On-site rescue and emergency call; died after hospital rescue.	Mainstream media source
4	2016-11-19	Physical fitness testing	Not disclosed	Wuhan University	Physical fitness test	Sudden collapse during testing followed by death despite rescue	Treated on site and transferred to hospital, where death was later confirmed.	Aggregated web source / public report
5	2017-02-17	Extracurricular campus sports	Male	Guangxi University	Informal basketball on campus	Sudden death	University issued notice and assisted with follow-up arrangements.	Mainstream media source / institutional source
6	2017-09-10	Extracurricular campus sports	Male	Fuzhou Institute of Technology	Playing basketball on campus	Initially identified as sudden death	Campus clinic response and hospital transfer; died despite rescue.	Local news source
7	2018-01-04	Extracurricular campus sports	Male	Zhejiang Institute of Mechanical and Electrical Engineering	Playing badminton	Sudden death	Died despite rescue after transfer to hospital.	Aggregated web source / public report
8	2018-04-26	Physical fitness testing	Not disclosed	Tianjin University	Physical fitness test	No explicit medical cause disclosed	Sudden collapse during testing and death despite rescue.	Aggregated/web-based public source / online news source
9	2019-03-21	Extracurricular campus sports	Male	Foshan University	Running on campus track	Cardiac arrest	On-site first aid and intensive hospital rescue; later brain death.	Mainstream media source
10	2019-10-19	Physical fitness testing	Male	Lushan College of Guangxi University of Science and Technology	1000-meter physical fitness test	No explicit medical cause disclosed	On-site treatment; died despite hospital rescue; police involved.	Aggregated web source / indexed public report
11	2019-10-27	Physical fitness testing	Male	Xuzhou Medical University	1000-meter running fitness test	Hospital diagnosis: sudden death	Continuous chest compressions on site; hospital transfer; rescue unsuccessful.	Mainstream media source
12	2019-11-09	Extracurricular campus sports	Female	Inner Mongolia University of Finance and Economics	Running on west campus track	Sudden death	University statement and campus safety follow-up.	Aggregated web source / public report
13	2021-04-29	Extracurricular campus sports	Male	Baise University	Collapse near basketball court	Family cited hospital diagnosis of cardiogenic sudden death	Hospital transfer and ICU admission; died later.	Official / institutional source
14	2021-11-27	Physical fitness testing	Male	Donghua University	Collapse after 1000-meter physical fitness test	Sudden death / death despite rescue	On-site rescue; university suspended subsequent fitness testing.	Local news source / public report
15	2022-03-24	During physical education class	Female	Shanxi University of Finance and Economics	Group jogging warm-up	Death confirmed by university notice	Teacher rescue, school hospital arrival, emergency transfer; working team established.	Aggregated web source / indexed public report
16	2022-09-15	Extracurricular campus sports	Male	Wuchang University of Technology	Playing basketball	Hospital diagnosis: respiratory and cardiac arrest	Transferred for emergency treatment; rescue unsuccessful.	Mainstream / online news source
17	2023-05-15	During physical education class	Male	A university in Nanchang	Sudden collapse while running	Preliminary diagnosis: cardiogenic sudden death	Emergency rescue unsuccessful; university denied fault.	Aggregated web source / indexed public report
18	2023-09-03	Extracurricular campus sports	Male	Guiyang Institute of Information Technology	Playing basketball	Preliminary diagnosis: sudden death (possibly cardiac)	Died despite emergency rescue by 120.	Official / institutional source
19	2024-04-12	Pre-class / morning exercise setting	Female	Baicheng Medical College	Sudden collapse before morning exercise	Underlying congenital heart disease; death related to underlying disease	On-site first aid, emergency call, hospital transfer; relevant personnel later held accountable.	Mainstream media source / investigation-related report
20	2024-06-09	Pre-competition campus training	Male	Shenyang Normal University	Weight-cutting training before boxing competition	Preliminary diagnosis: sudden death	Collapsed in dormitory; emergency personnel attempted rescue for about half an hour.	Aggregated web source / public report
21	2025-04-02	Campus competition / selection event	Male	Henan Mechanical and Electrical Vocational College	Running event in sports meet selection contest	Publicly described as cardiogenic sudden death	Lost consciousness during race; no vital signs when rescue personnel arrived.	Official / institutional source

This table summarizes 21 publicly reported cases included in the main analytical sample. Cause-of-death descriptions are reproduced from public reports and should not be interpreted as independent clinical determinations by the author. Response information refers to publicly reported on-site aid, emergency activation, hospital transfer, or related follow-up actions. Detailed source platforms, URLs, and source quality notes are provided in Additional file 1. Case 20 was retained as a boundary-included case because the public report explicitly linked the collapse to weight-cutting training before a university boxing competition; however, it was interpreted cautiously because the collapse was reported to have occurred in a dormitory after the training context

**Table 3:** Supplementary successful rescue cases

Case ID	Date	University	Setting Category	Core Description	Emergency Measures	Outcome	Remarks
R1	2020-01-15	Suzhou University	Physical education / running	A student experienced sudden cardiac arrest while running.	Teachers, students, and school medical staff jointly performed CPR and AED defibrillation.	Successfully rescued and out of danger.	Not included in the fatal-case main sample.
R2	2020-11-17	Nanjing University of Aeronautics and Astronautics	Physical fitness testing	A female student experienced sudden cardiac arrest during an 800-meter fitness test.	Physical education teachers, school medical staff, and emergency services provided relay rescue for approximately 36 min.	Vital signs were restored.	Not included in the fatal-case main sample.
R3	2023-03-10	A university in Chengdu	Running exercise	A student experienced sudden cardiac arrest while running during a cold/flu illness.	Rescue was carried out in coordinated succession at the hospital.	Successfully treated.	Diagnosed with fulminant myocarditis.
R4	2023-08-28	Sun Yat-sen University, Shenzhen Campus	Classroom setting	A medical student experienced sudden cardiac arrest in class.	Teachers and students used an AED and performed CPR.	Out of danger.	Not a sports setting, but a successful rescue case involving a university student.
R5	2024-05-15	Zhejiang University City College	Activity on sports ground	A student collapsed on the sports ground and developed respiratory and cardiac arrest.	Fellow students performed relay CPR and AED defibrillation.	Spontaneous breathing was restored and the student survived.	Successful rescue case on a campus sports ground.
R6	2024-09	Zhejiang Sci-Tech University College of Science and Art	Ball-game activity	A student experienced cardiac arrest while playing ball.	CPR and AED emergency treatment were administered on site.	Ultimately survived.	Source documentation may be further strengthened.
R7	2024-10-27	A university in Shanxi	1000-meter fitness test	A first-year student developed respiratory and cardiac arrest during a 1000-meter fitness test.	On-site first aid, assisted ventilation, intravenous access, and medication were administered.	Heartbeat recovered in approximately 3 min.	Successful rescue case in a fitness-testing setting.
R8	2024-11	Nanjing University of Posts and Telecommunications	1000-meter fitness test	A student suddenly collapsed after a fitness test.	Physical education teachers and fellow students used an AED and performed CPR.	Successfully revived.	It is advisable to add the university’s official social media post or the provincial Red Cross original link later.
R9	2025-05	Hohai University, Jiangning Campus	Basketball-court activity	A senior student experienced sudden cardiac arrest while playing basketball.	Fellow students, the campus hospital, and emergency services provided relay rescue.	Successfully survived.	Successful rescue case in a campus sports setting.

The cases listed in this table involved university students who experienced sudden cardiac arrest or respiratory and cardiac arrest in sports activities, physical fitness testing, classroom, or campus settings but survived after emergency response. They were not included in the fatal-case main analytical sample and were used only as contextual illustrations for interpreting emergency preparedness

### Thematic analysis and coding procedure

Thematic analysis was conducted using a reflexive and inductive approach informed by Braun and Clarke’s framework [6, 7]. To enhance coding transparency, five trained coders assisted with preliminary coding of the public case materials. The coders had backgrounds in physical education, sports science, or school health-related research and were briefed on the study aim, inclusion boundaries, source limitations, and coding principles before coding.

The analysis followed a staged procedure. First, the author developed the initial extraction framework and coding principles based on the research questions and repeated reading of the included case files. Second, the five coders independently reviewed the case materials and generated preliminary open codes concerning reported health vulnerability, activity type, exercise load, warning signs, screening and monitoring information, student behavior, psychosocial or evaluative context, environmental conditions, emergency response, and institutional follow-up. Third, the coding outputs were compared and synthesized by the author. Coding inconsistencies and ambiguous excerpts were discussed with the coders, and unresolved issues were adjudicated by the author with reference to the original public reports and the operational definitions in the codebook.

Fourth, related codes were grouped into intermediate categories. Fifth, these categories were compared across cases to identify recurrent reporting-based patterns. Finally, candidate themes were reviewed against the original case materials and refined to reflect contextual patterns visible in public reports rather than causal risk factors. The final category construction, theme development, theme naming, interpretation of findings, and manuscript writing were completed by the author.

Because the study adopted reflexive thematic analysis rather than a positivist content analysis design, formal inter-coder reliability statistics such as Cohen’s kappa were not calculated. Coding comparison and discussion were used to enhance analytic transparency, challenge initial interpretations, and improve auditability rather than to claim statistical reproducibility.

The codebook was used as an audit tool throughout coding comparison and theme refinement. It specified code names, operational definitions, inclusion rules, exclusion rules, and illustrative examples. To integrate the codebook more directly into the manuscript, the Results section includes a summary table linking each higher-order theme with its corresponding intermediate categories, representative codes, and case-based examples. The complete codebook is provided as Additional file 2.

Through this process, six higher-order themes were developed: individual health vulnerability; mismatch between task demands and physical preparedness; limited visibility of health screening and classroom monitoring; incomplete public reporting of emergency preparedness and on-site rescue capacity; evaluative contexts and possible barriers to symptom reporting; and the amplifying role of environmental conditions.

### Credibility, reflexivity, and auditability

Several measures were used to enhance credibility and analytic transparency. First, multiple reports of the same event were cross-checked whenever possible, and priority was given to official notices and more authoritative or information-rich sources. Second, a structured extraction framework and codebook were applied consistently across included cases. Third, five trained coders assisted with preliminary coding, and discrepancies or ambiguous coding decisions were reviewed through discussion. Fourth, uncertain or incomplete information was explicitly recorded as “not reported” or “unclear” rather than inferred. Fifth, the author repeatedly returned to the original source materials during code comparison, category construction, and theme refinement to ensure that the themes remained grounded in the available public record.

Because the study adopted reflexive thematic analysis, coding comparison was used to improve analytic transparency rather than to produce a statistical measure of inter-coder agreement. Formal inter-coder reliability statistics were not calculated. An audit trail was maintained, including search terms, case selection decisions, extraction fields, coding categories, codebook revisions, theme development, and source limitations. The purpose of this audit trail was to make the analytic process transparent and reviewable, not to claim statistical reproducibility.

A reflexive stance was maintained throughout the analysis. Particular care was taken to avoid over-attributing individual deaths to any single factor in the absence of verified medical records, autopsy findings, emergency response logs, or institutional investigation files. The themes were therefore interpreted as recurrent public-record patterns that may inform prevention-oriented discussion, rather than as confirmed causal mechanisms.

### COREQ applicability

The Consolidated Criteria for Reporting Qualitative Research (COREQ) [8] was used as a transparency-oriented reporting reference rather than as a full design-specific checklist, because this study did not involve interviews, focus groups, participant recruitment, interviewer-participant relationships, audio-recording, transcript generation, or participant checking. Therefore, COREQ items concerning interviewer characteristics, participant selection, interview setting, transcription, and participant feedback were not applicable.

The applicable principles of COREQ were operationalized in three areas: (1) transparency of research design and data source description; (2) clarity of coding, theme development, and analytic reflexivity; and (3) explicit reporting of limitations related to source reliability, data completeness, and transferability.

A brief COREQ applicability table is provided in Additional file 3 to distinguish applicable, partially applicable, and non-applicable items. The checklist was used to improve transparency in reporting the qualitative analytic process, not to imply that the study involved interview-based qualitative data.

### Methodological boundaries

This study has several methodological boundaries that are important for interpretation. Publicly available case reports are not equivalent to clinical records, surveillance data, emergency medical service records, or institutional investigation files. They are shaped by newsworthiness, public disclosure practices, family attention, institutional communication, search engine indexing, and media framing. Therefore, the cases included in this study should be understood as cases that became visible in the public record rather than as a complete or representative sample of all non-traumatic sudden deaths among university students in China.

Public reports may simplify event processes, omit key clinical or institutional details, or reproduce partial narratives from different stakeholders. For this reason, the analysis distinguished between confirmed information reported by public sources, uncertain or inconsistent information, and information that was not reported. Information that was absent from public reports was not treated as evidence that a procedure, condition, or event did not exist.

The dataset also lacks denominator information, such as the total number of university students exposed to physical education classes, physical fitness testing, campus competitions, training sessions, or extracurricular campus sports during the study period. It therefore cannot support incidence estimation, risk comparison across activity types, or statistical assessment of exposure-outcome relationships.

Accordingly, the findings of this study should be interpreted as public-record-based contextual patterns and reporting-based contextual signals. They cannot establish medical causality, incidence, prevalence, institutional responsibility, or statistically verifiable risk factors. Their value lies in showing what types of contextual information become visible in public reports and how these reported patterns may inform hypothesis generation, prevention-oriented communication, and future empirical research on campus sports safety governance.

### Ethical considerations

This study used only publicly accessible web-based materials and did not involve direct interaction with human participants. No private interviews, surveys, medical records, emergency medical service records, or institutional confidential files were collected or analyzed.

Because the cases involved deceased students and sensitive family and institutional contexts, the analysis followed a minimization principle. Personal names, student identification information, family details, and unnecessary biographical information were not extracted or reproduced. Case descriptions were summarized at the event and contextual level, and the analysis focused on public-record-based patterns rather than individual blame, institutional accusation, or independent clinical diagnosis.

The research was conducted in accordance with the ethical principles of the Declaration of Helsinki. Because this study was based on secondary analysis of publicly available materials and did not involve private data collection or direct human participation, ethics committee approval and informed consent were not required.

## Results

### Basic case characteristics and source profile

This section presents the reporting-based findings from the 21 included fatal cases. The results are organized around six higher-order themes developed through thematic analysis: individual health vulnerability; mismatch between task demands and physical preparedness; limited visibility of health screening and classroom monitoring; incomplete public reporting of emergency preparedness and on-site rescue capacity; evaluative contexts and possible barriers to symptom reporting; and the amplifying role of environmental conditions.

The case-based examples below illustrate how specific contextual information was described in public reports. They should not be read as evidence that any single factor caused death in an individual case. Because the public materials varied in completeness, the examples are treated as public-record-based contextual signals rather than clinically verified mechanisms.

Two paraphrased examples illustrate the type of public material used for coding. In one fitness-testing case, public reports described a student who collapsed during a 1000-meter physical fitness test and later died despite emergency treatment; the reports did not provide a complete pre-test screening record or AED timeline. In another case, public materials described continuous chest compressions and hospital transfer after a collapse during a campus sport activity, while the full emergency-response timeline remained unclear. These examples were used to illustrate how contextual information appeared in public reports, not to infer clinical causality or institutional responsibility.

A total of 21 publicly reported fatal cases of non-traumatic sudden death among university students in physical education classes and campus sports-related settings between 2016 and 2025 were included in the main analytical sample. These incidents appeared sporadically in the public record over the study period. Because the dataset was derived from public reports rather than systematic surveillance, this temporal distribution should be interpreted only as continued public visibility of such events, not as an incidence trend or evidence of increasing or decreasing risk.

The included cases covered a range of campus exercise contexts, including physical education classes, physical fitness testing, campus competitions, selection events, extracurricular exercise, pre-competition training, and pre-class or morning-exercise-related settings. Running- and endurance-related activities appeared frequently in the public reports, including 800-meter races, 1000-meter physical fitness tests, group jogging, and campus track running. Ball-game contexts, such as basketball and badminton, were also represented. Among cases in which sex was publicly identifiable, male students predominated; however, sex was not consistently reported across all cases.

Cause-of-death descriptions varied across reports and commonly included sudden death, cardiogenic sudden death, respiratory and cardiac arrest, cardiac arrest, or death after unsuccessful rescue. These descriptions were recorded as reported by public sources and were not treated as independent clinical determinations by the author. Most cases mentioned some form of rescue effort, emergency call, school medical staff involvement, hospital transfer, or institutional follow-up, but the completeness of emergency-response information varied substantially.

Table 1 clarifies the boundary between included cases and excluded student safety incidents. Table 2 summarizes the 21 fatal cases included in the main analysis. Table 3 provides supplementary successful rescue cases, which were not included in the fatal-case analytical sample but were used to contextualize emergency preparedness. Additional file 1 provides the case-level source inventory and source quality notes; Additional file 2 provides the full codebook; and Additional file 3 explains the applicability of COREQ items to this public-document-based qualitative study.

Table 4 summarizes the main source types and source-quality profile of the 21 included fatal cases. The source profile indicates that the public record was heterogeneous: some cases were based on official or institutional statements, some on mainstream or local media reports, and others on aggregated or indexed public reports. This variation reinforced the need to interpret the findings as patterns visible in public reporting rather than as verified epidemiological or clinical evidence.

**Table 4:** Summary of source types and source quality among the 21 included fatal cases

Source type	Number of cases	Main source basis	Source-quality interpretation
Official or institutional source	3	University notice, institutional response, official investigation-related report	Higher source authority and closer proximity to the event
Mainstream or local media source	6	Established national, provincial, or local media report	Moderate to high authority; often provides contextual details
Aggregated or indexed public report	9	Reposted, indexed, or aggregated web-based public report	Used cautiously because completeness, proximity, and attribution may vary
Mixed-source case file	3	Combination of official, institutional, media, and follow-up reports	Used for cross-checking and contextual supplementation
Total	21	-	-

Source type was classified according to the main source basis used for each case file. When multiple reports were available for the same incident, official or institutional sources were prioritized, and other reports were used only for cross-checking or contextual supplementation. Source-quality interpretation was based on source authority, proximity to the event, information completeness, cross-source consistency, and transparency of event description

Table 5 links the six higher-order themes to intermediate categories, representative codes, and case-based examples. This table is intended to make the analytic pathway more transparent by showing how public-record information was coded and grouped into themes.

**Table 5:** Linkage between higher-order themes, intermediate categories, representative codes, and case-based examples

Higher-order theme	Intermediate category	Representative codes	Example source information	Interpretive boundary
Individual health vulnerability	Reported or suspected vulnerability	underlying disease; cardiogenic description; health unclear	prior congenital heart disease; reported cardiac arrest	Not independent clinical diagnosis
Task demands and preparedness	High-load or performance task	endurance test; running; ball game; training	1000-m test; 800-m race; basketball; training	Activity type is contextual, not causal
Screening and monitoring	Unclear risk identification	screening not reported; warning signs unclear	collapse reported; pre-test screening unclear	Not reported does not mean absent
Emergency preparedness	Incomplete rescue-chain information	emergency call; CPR; AED unclear	rescue mentioned; AED timing unclear	Rescue quality cannot be verified
Evaluative context	Assessment or competition pressure	fitness test; race; selection; competition	1000-m test; selection contest	Possible context, not psychological proof
Environmental conditions	Background exposure	morning exercise; outdoor running; weather unclear	outdoor or morning activity reported	Not an independent cause

### Individual health vulnerability and reported underlying conditions

Individual health vulnerability was one of the recurrent themes visible in the public case materials. In this study, it refers to reported underlying disease, suspected latent health risk, previously abnormal signs, insufficient physical readiness, or other physiological vulnerabilities that may have affected students’ capacity to tolerate exercise-related demands. Analytically, this theme was used to capture how health vulnerability was represented, or left unclear, in the public record; it was not used to assign clinical causality.

A relatively information-rich example was the Baicheng Medical College case, in which the public investigation-related report mentioned a prior diagnosis of congenital heart disease and linked the death to the underlying condition. This case was coded under “known underlying disease” and contributed to the intermediate category of reported individual vulnerability. In contrast, several other cases were publicly described as cardiogenic sudden death, cardiac arrest, respiratory and cardiac arrest, or sudden death, but public materials did not provide detailed pre-event medical histories, autopsy findings, or longitudinal health information.

Across the included cases, the public record showed three subtheme-level patterns. First, some reports mentioned known or suspected underlying conditions. Second, many reports described sudden collapse or loss of consciousness without sufficient information on prior symptoms or health status. Third, health vulnerability was often visible only after the fatal event, through post-event descriptions, hospital diagnoses, or institutional responses.

These patterns suggest that public reports sometimes make individual vulnerability visible, but they rarely provide enough evidence to reconstruct students’ actual health status before participation. Therefore, this theme should be interpreted as a reporting-based signal that highlights the importance of health communication and pre-participation documentation, not as proof that underlying disease caused the fatal outcomes in all cases.

### Mismatch between task demands and physical preparedness

Mismatch between task demands and physical preparedness emerged as another recurrent contextual theme. It captures public-record descriptions in which the content, intensity, completion mode, or evaluative nature of physical education classes, physical fitness tests, competitions, selection activities, or training may not have been clearly matched to differences in students’ health status, fitness base, exercise habits, or immediate physical condition.

Running and endurance-related activities were frequently represented in the main analytical sample. Examples included 800-meter race events, 1000-meter physical fitness tests, group jogging during physical education classes, and campus track running. Ball-game activities, such as basketball and badminton, were also represented. One boundary-included case involved weight-cutting training before a university boxing competition and was interpreted cautiously because the collapse was reported to have occurred in a dormitory after the training context.

These cases illustrate that publicly reported fatal incidents occurred across several activity types involving different degrees of cardiopulmonary demand, physical challenge, competition, or training pressure. The theme does not imply that these activities are inherently unsafe or that they directly caused death. Rather, the recurrent reporting pattern suggests that future institutional documentation and empirical research should pay closer attention to whether exercise tasks are implemented with appropriate differentiation, temporary adjustment, exemption, withdrawal, and monitoring mechanisms.

In coding terms, examples such as “collapse during 1000-meter physical fitness test,” “collapse during 800-meter race,” “group jogging warm-up,” and “pre-competition training” were grouped under activity demand and task-load-related codes. These codes contributed to the intermediate category of high cardiopulmonary or performance-related task demand and then to the final theme of mismatch between task demands and physical preparedness.

### Limited visibility of health screening and classroom monitoring

Limited visibility of health screening and classroom monitoring constituted a major recurring public-record-based theme. This theme refers not to confirmed absence of screening or monitoring, but to the limited information available in public reports about pre-exercise risk identification, stratified management, teacher observation, warning-sign recognition, temporary withdrawal procedures, and intervention before collapse.

In several physical fitness testing cases, public reports described students collapsing during or shortly after 1000-meter or other running tests, but did not report whether individualized pre-test risk stratification, temporary exemption procedures, or prior symptom-reporting mechanisms had been implemented. Similarly, in cases involving physical education classes, group jogging, basketball, badminton, or training, reports often described sudden collapse or loss of consciousness, while providing little information about whether warning signs had appeared before collapse or whether teachers, classmates, or medical staff had observed earlier abnormalities.

The Baicheng Medical College case was comparatively information-rich because public materials mentioned an underlying congenital heart disease. However, most cases did not provide comparable detail about pre-exercise health files, medical histories, family histories, recent illness, prior symptoms, or teacher monitoring records. This contrast illustrates that the visibility of screening and monitoring information varied substantially across public reports.

Three subtheme-level patterns were identified. First, pre-exercise health screening was often not described in public materials. Second, classroom or field monitoring processes before collapse were usually unclear. Third, the public record rarely clarified whether differentiated management, alternative assessment, or temporary withdrawal options had been activated.

These findings should not be interpreted as evidence that screening or monitoring was absent in all cases. Rather, they show that such information was often not visible in public reports. This limited visibility itself is important, because it suggests that future campus sports safety documentation should make risk identification, task adjustment, monitoring, and response procedures more transparent and reviewable.

### Incomplete public reporting of emergency preparedness and on-site rescue capacity

Incomplete public reporting of emergency preparedness and on-site rescue capacity emerged as another recurrent theme. Emergency preparedness refers to the availability and coordination of early recognition, emergency activation, cardiopulmonary resuscitation, AED access, trained responders, emergency transfer, and post-event review. Because public reports rarely provided complete rescue timelines or equipment details, this theme was coded as incomplete public reporting rather than as confirmed absence of emergency capacity. This concern is consistent with recent attention to sports-related sudden cardiac arrest in children and adolescents [9].

Most fatal cases mentioned some degree of rescue effort, such as on-site first aid, calling emergency services, school clinic or medical staff involvement, hospital transfer, or unsuccessful hospital rescue. However, public materials varied considerably in the amount of emergency-response information provided. In some cases, reports mentioned chest compressions, emergency calls, or transfer to hospital; in other cases, they only stated that the student died despite rescue.

For example, in the Xuzhou Medical University case, public reports mentioned continuous chest compressions on site and hospital transfer. In other cases, such as some physical fitness testing, basketball, or campus competition cases, reports mentioned emergency call or hospital rescue but did not clarify whether an AED was immediately available, how quickly CPR was initiated, whether responders were systematically trained, or how long emergency medical services took to arrive. This variation illustrates that emergency response was often visible in public reports, but the completeness of the rescue chain was not consistently documented.

Supplementary successful rescue cases provide contextual illustrations for interpreting this theme. These cases were not included in the fatal-case main analytical sample, but they show how timely CPR, AED use, relay rescue, and coordinated response were described in public reports of survival. Therefore, Table 3 was used only as supplementary contextual evidence for emergency preparedness, not as direct comparative evidence against the fatal cases.

Overall, the reporting pattern suggests that emergency preparedness should be documented as a response-chain issue rather than as a single-equipment issue. The public record often mentioned rescue efforts, but it rarely provided enough information to evaluate the full chain from early recognition to emergency activation, CPR quality, AED use, emergency medical service arrival, hospital transfer, and post-event review.

### Evaluative contexts and possible barriers to symptom reporting

Evaluative contexts and possible barriers to symptom reporting also emerged as a recurrent contextual theme. In this study, evaluative context refers to physical education classes, physical fitness tests, sports-meet events, selection activities, competitions, or training situations in which students may perceive pressure related to grades, credits, performance, peer expectations, institutional requirements, or competition outcomes.

Several included cases occurred in settings with an evaluative or competitive character, including 1000-meter physical fitness tests, 800-meter race events, sports-meet selection contests, and collective physical education activities. These settings may shape how discomfort is interpreted and reported, because students may feel pressure to complete a task, avoid delaying the group, protect academic or assessment outcomes, or conform to peer and institutional expectations.

Public reports did not usually provide direct evidence of students’ subjective decision-making before collapse. Therefore, this theme does not claim that evaluative pressure caused the fatal events. Rather, it identifies a contextual reporting pattern: some incidents occurred in settings where task completion, assessment, or competitive participation may have made symptom reporting and temporary withdrawal more difficult.

In coding terms, cases involving “physical fitness testing,” “1000-meter running test,” “800-meter race,” “selection contest,” or “pre-competition training” were coded as evaluative or performance-related exercise settings when the public record indicated an assessment, selection, competition, or training context. These codes were grouped into the intermediate category of performance or assessment pressure and contributed to the final theme of evaluative contexts and possible barriers to symptom reporting.

This theme suggests that future campus sports safety research should examine not only whether students have known disease or whether exercise intensity is high, but also whether students know when to stop, whether they feel permitted to report discomfort, whether temporary withdrawal is non-punitive, and whether alternative assessment or task-adjustment mechanisms are clearly communicated. Athlete mental health research has identified multiple barriers and facilitators to help-seeking [10]. Qualitative and sport-specific studies further suggest that stigma, performance expectations, and uncertainty about support pathways may shape whether athletes disclose difficulties or seek help [11, 12]. A recent scoping review has also highlighted variation in athletes’ access to and experiences of help-seeking support [13]. Related research on youth athlete concussion has shown that under-reporting decisions may be influenced by social, competitive, and contextual factors [14].

### The amplifying role of environmental conditions

The amplifying role of environmental conditions constituted the sixth contextual theme. Environmental conditions include seasonal variation, temperature fluctuation, early-morning activity periods, weather conditions, air quality, and other external exposures that may affect physiological load and adaptive capacity during exercise.

Public reports did not consistently provide detailed environmental information. Some cases occurred in settings where environmental context was mentioned or could be partially inferred from the reported timing, such as morning exercise, pre-class activity, outdoor physical fitness testing, campus competitions, or running on campus tracks. However, in many cases, public materials did not provide precise temperature, humidity, air quality, venue condition, or weather data.

This theme therefore should not be interpreted as evidence that environmental conditions independently caused the fatal events. Rather, environmental conditions were coded when they appeared in the public record as possible background or interacting exposures. They may interact with individual health vulnerability, task demands, psychological tension, and on-site safeguards, but the public materials were not sufficient to verify such interactions clinically or statistically.

The reporting pattern suggests that environmental information should be more systematically documented in future campus sports safety records. For prevention-oriented governance, environmental conditions may need to be considered alongside health screening, task stratification, classroom and field monitoring, symptom reporting, and emergency preparedness.

## Discussion

This study synthesized 21 publicly reported fatal cases of non-traumatic sudden death among university students in physical education and campus sports-related settings in China between 2016 and 2025. Six recurrent reporting-based contextual themes were identified: individual health vulnerability; mismatch between task demands and physical preparedness; limited visibility of health screening and classroom monitoring; incomplete public reporting of emergency preparedness and on-site rescue capacity; evaluative contexts and possible barriers to symptom reporting; and the amplifying role of environmental conditions.

The central contribution of the study is not to establish epidemiological risk factors, clinical causality, or institutional responsibility. Rather, it is to organize the contextual signals that became visible in public records and to discuss how these signals may inform hypothesis generation, prevention-oriented communication, and campus sports safety governance.

### What the data can and cannot support

Publicly available case reports cannot support causal inference, incidence estimation, or statistical association. The 21 cases included in this study should therefore be understood as incidents that became visible in the public record, rather than as a complete or representative sample of all relevant events. The dataset lacks denominator information and does not include verified clinical records, emergency response logs, or internal institutional investigation files.

What the data can support is a qualitative synthesis of public-record-based contextual signals, including reported settings, activity demands, health information, monitoring visibility, evaluative contexts, emergency-response descriptions, and environmental information. These signals provide a structured basis for hypothesis generation and future empirical research, while remaining distinct from confirmed causal mechanisms or epidemiological risk patterns.

### A systems-oriented conceptual framework

The six themes identified in this study can be interpreted through a socio-ecological and systems-oriented perspective on campus sports safety. From this perspective, publicly reported fatal incidents should not be reduced to a single exercise task, a single medical condition, or a single institutional procedure. Instead, they can be understood as events represented within layered contexts involving individual vulnerability, activity demands, institutional monitoring, psychosocial pressures, environmental exposure, and emergency-response capacity.

Conceptually, the six themes form a reporting-based risk-context framework rather than a verified causal chain. Individual health vulnerability and task demands describe possible pre-event and event-level contexts visible in public reports. Screening, classroom monitoring, and symptom reporting describe institutional and psychosocial processes through which warning signs may or may not become visible. Environmental conditions may operate as background exposures that interact with physiological load. Emergency preparedness describes the post-collapse response capacity that becomes relevant once a critical event occurs.

**Fig. 2:**
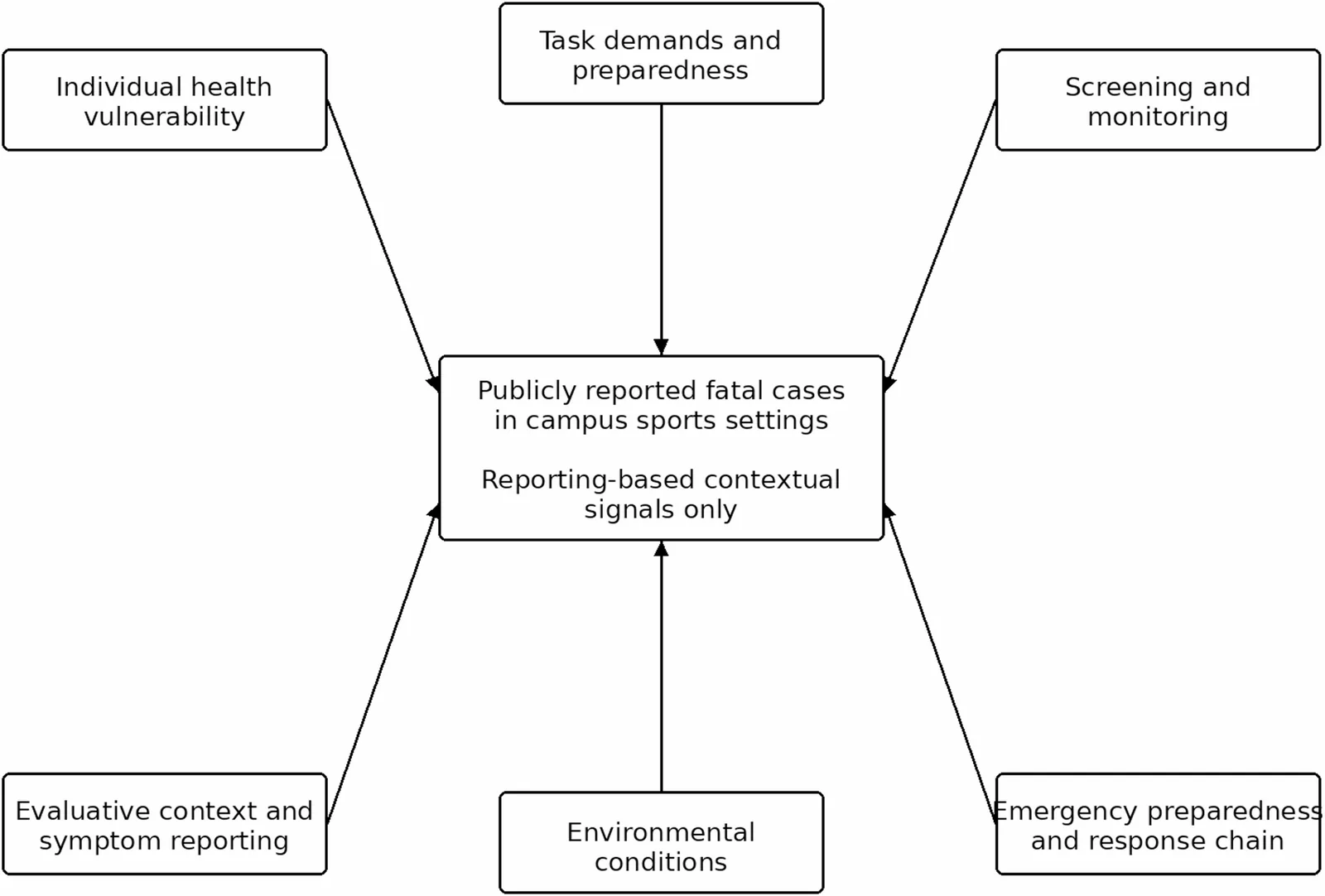
Conceptual framework of public-record-based contextual dimensions in campus sports-related sudden death reports

Figure 2 summarizes these six contextual dimensions as an exploratory public-record-based framework for campus sports safety reflection. The framework does not imply that the six dimensions occurred in every case, nor does it suggest that they caused the deaths. Instead, it shows how publicly reported information can be organized into a prevention-oriented structure: upstream health communication and screening, risk-sensitive task adjustment, real-time monitoring and symptom reporting, environmental risk awareness, and emergency-response readiness.

From this systems-oriented perspective, prevention in campus sports settings does not depend on a single intervention. Rather, it requires coordination among health communication, task stratification, teacher observation, student symptom-reporting norms, environmental risk assessment, AED accessibility, CPR training, emergency action planning, and post-event review. This conceptual synthesis remains exploratory because it is derived from public reports, but it provides a structured way to translate reporting-based patterns into questions for future empirical research and practical governance improvement.

### Implications for prevention-oriented campus sports safety governance

The findings point to several areas for prevention-oriented institutional review in campus sports safety governance. These implications should be understood as practice-oriented reflections rather than conclusions about confirmed causal risk factors.

First, universities may consider moving risk communication upstream. Public reports often provided limited information about pre-exercise screening, previous symptoms, known disease history, recent illness, or temporary contraindications to exercise. This suggests the importance of clearer mechanisms for students to report known health conditions, recent discomfort, illness, sleep deprivation, medication use, or other temporary states that may affect exercise tolerance. Such mechanisms should not be limited to one-time health forms; they may need to operate continuously across physical education classes, fitness testing, competitions, and extracurricular campus sports.

Second, task stratification and adjustment mechanisms deserve closer attention. Running, physical fitness testing, ball games, selection events, and pre-competition training were frequently represented in the public reports. These activities are not inherently inappropriate. However, their implementation may need to account for students’ health status, fitness base, exercise habits, and immediate physical condition. Universities may therefore consider clearer procedures for differentiated task design, temporary exemption, alternative assessment, task withdrawal, and return-to-participation decisions.

Third, symptom reporting should be made safe, visible, and non-punitive. Some cases occurred in evaluative or competitive contexts, such as 1000-meter fitness tests, 800-meter races, selection contests, and collective physical education activities. Public reports did not provide direct evidence of students’ subjective decision-making, but they suggest that symptom reporting and temporary withdrawal are important governance issues in assessment-oriented sports settings. Students should know what warning signs require stopping, whom to report to, and whether withdrawal will lead to unfair penalty or stigma.

Fourth, classroom and field monitoring should be documented as a process rather than assumed as a background condition. Public reports often described sudden collapse but provided limited information about warning signs, teacher observation, peer response, or temporary task adjustment before collapse. For prevention-oriented governance, monitoring may need to include pre-activity confirmation, observation during high-load tasks, clear stop signals, peer alert mechanisms, and post-incident documentation.

These implications point toward a governance approach that connects health screening, task adjustment, symptom reporting, teacher monitoring, and student education. The goal is not to restrict ordinary physical education or campus sport participation, but to make participation safer, more responsive, and better matched to individual and contextual conditions.

### Emergency preparedness and response-chain improvement

Emergency preparedness deserves particular attention because it shapes the possibility of intervention after collapse has occurred. In the fatal cases included in this study, most public reports mentioned some form of rescue effort, emergency call, hospital transfer, or institutional response. However, many reports did not clarify whether an automated external defibrillator was immediately available, whether trained responders initiated high-quality cardiopulmonary resuscitation at the earliest possible moment, how quickly emergency services arrived, or whether a rehearsed emergency action plan was activated.

The supplementary successful rescue cases provide contextual illustrations of why the emergency response chain matters. These cases were not analyzed as part of the fatal-case sample and should not be treated as direct comparative evidence. Nevertheless, they show that public reports of survival often highlighted timely CPR, AED use, relay rescue, and coordinated response. This contrast suggests that future campus sports safety research should collect more complete emergency-response data, including collapse-to-recognition time, collapse-to-CPR time, collapse-to-AED time, AED availability, responder training, emergency medical service arrival time, and post-event review procedures.

From a public health and campus governance perspective, emergency preparedness in athletic settings has been emphasized in consensus recommendations on sudden cardiac arrest management in high school and college athletic programs [15]. Evidence on exercise-related sudden cardiac arrest survival and bystander intervention further highlights the importance of early recognition, rapid CPR, and coordinated response [16, 17]. Broader preparedness guidance has also discussed strategies to optimize response systems [18], the design and implementation of emergency action plans [19], school-based AED programs [20], emergency cardiac care across athletic settings [21], and the role of AEDs in sport [22]. These settings include tracks, gymnasiums, stadiums, fitness-testing sites, basketball courts, and other campus exercise venues. Physical education teachers, coaches, school medical staff, security personnel, student volunteers, and emergency service providers should be considered part of a coordinated response chain rather than isolated actors.

Emergency preparedness should therefore be understood as a response-chain issue, not merely as an equipment issue. A functional response chain requires early recognition, immediate emergency activation, rapid CPR, accessible AED use, coordinated transfer, and post-event review. Because public reports rarely provide complete emergency timelines, future studies should incorporate emergency medical service records, institutional emergency logs, and interviews with responders to examine how this chain operates in real campus settings.

### Limitations and future research

This study has several limitations. It relied on publicly reported materials rather than verified medical records, autopsy findings, emergency medical service records, or internal institutional investigation files. Public reporting is also subject to visibility and selection bias, and the completeness of information varied across cases. Missing information on screening, symptoms, AED availability, response timing, or environmental conditions was therefore treated as “not reported” rather than as evidence that these elements were absent.

The dataset lacks denominator data and cannot estimate incidence, compare risk across exercise contexts, or identify statistically confirmed risk factors. Although five trained coders assisted with preliminary coding and coding discussion, final category construction, theme development, interpretation, and writing were completed by the author. Because the study used reflexive thematic analysis, formal inter-coder reliability statistics were not calculated. The findings are also based on publicly available Chinese-language materials from mainland China and may not be directly transferable to other settings.

Future research should incorporate stronger and more diverse data sources, including medical documentation, emergency response records, institutional safety files, physical education management records, interviews with teachers and responders, and student surveys on symptom reporting and withdrawal behavior. Prospective campus safety surveillance would help clarify exposure contexts, emergency-response timelines, AED coverage, first-aid training, and risk-stratified exercise management in real university settings.

## Conclusions

This study identified six recurrent public-record-based contextual themes in publicly reported cases of non-traumatic sudden death among university students in physical education and campus sports-related settings in China: individual health vulnerability; mismatch between task demands and physical preparedness; limited visibility of health screening and classroom monitoring; incomplete public reporting of emergency preparedness and on-site rescue capacity; evaluative contexts and possible barriers to symptom reporting; and the amplifying role of environmental conditions.

These findings should not be interpreted as evidence of causal risk factors, incidence, population-level association, or confirmed institutional responsibility. Rather, they represent reporting-based contextual signals visible in public records. The study contributes by organizing how such events are publicly described and by identifying areas that warrant further empirical investigation and prevention-oriented reflection.

From a campus sports safety governance perspective, the findings suggest several areas that universities may consider: clearer health communication before participation, risk-sensitive task stratification in physical education and fitness testing, improved classroom and field monitoring, non-punitive symptom reporting and temporary withdrawal mechanisms, attention to environmental conditions, and strengthened emergency-response systems involving AED accessibility, CPR training, emergency action plans, and routine drills.

Future research should incorporate stronger data sources, including medical documentation, emergency response records, institutional safety files, physical education management records, interviews with teachers and responders, and student surveys on symptom reporting and withdrawal behavior. Such work would help move beyond public-record-based reporting patterns toward a fuller understanding of vulnerability identification, exercise-task management, emergency preparedness, and prevention-oriented campus sports safety governance.

## Supplementary Information

Supplementary Material 1.

Supplementary Material 2.

Supplementary Material 3.

## Data Availability

The data supporting the conclusions of this article are derived from publicly accessible web-based materials and are included within this article and its additional files. The analytical case framework is presented in the main tables, and direct web links for the publicly reported cases included in the analysis are provided in Additional file 1, which lists the case-level source materials, website or platform names, URLs, source types, and source quality notes.No proprietary dataset was used. Because this study was based on secondary analysis of publicly available web-based materials rather than repository-deposited datasets, no accession numbers apply. To minimize unnecessary identification of sensitive cases, personal names, student identification information, family details, and unnecessary biographical information were not reproduced in the manuscript. No proprietary dataset was used. Because this study was based on secondary analysis of publicly available web-based materials rather than repository-deposited datasets, no accession numbers apply. To minimize unnecessary identification of sensitive cases, personal names, student identification information, family details, and unnecessary biographical information were not reproduced in the manuscript.

## Abbreviations

AED: Automated external defibrillator
CPR: Cardiopulmonary resuscitation
COREQ: Consolidated Criteria for Reporting Qualitative Research
