# Supplementary material for: Contextual patterns in publicly reported non-traumatic sudden deaths among university students in physical education and campus sports settings in China: a retrospective thematic analysis
Source: BMC Public Health. 2026 Jun 8;26:2683. doi: 10.1186/s12889-026-28079-5 (PMC13615593; doi:10.1186/s12889-026-28079-5)
Supplement: Supplementary file 1 [file 12889_2026_28079_MOESM1_ESM.docx]

**Additional file 3. COREQ applicability checklist**

Title: Applicability of COREQ items to a public-document-based retrospective thematic analysis

# 1. Purpose of this checklist

The Consolidated Criteria for Reporting Qualitative Research (COREQ) was originally developed for interviews and focus groups. The present study did not involve participant recruitment, interviews, focus groups, interviewer-participant relationships, audio-recording, transcription, or participant checking. Therefore, not all COREQ items were applicable.

This checklist explains how COREQ was used as a transparency-oriented reporting reference in the present public-document-based retrospective thematic analysis. Items were classified as applicable, partially applicable, or not applicable. For applicable and partially applicable items, the corresponding location in the manuscript or supplementary materials is indicated.

# 2. COREQ applicability table

Table A3.1. COREQ applicability checklist for this study

| **COREQ domain/item** | **Applicability to this study** | **How addressed in this manuscript** |
| --- | --- | --- |
| Domain 1: Research team and reflexivity | Partially applicable | This manuscript has one listed author. Five trained coders assisted with preliminary coding of the public case materials, while the author integrated the coding outputs and conducted the final theme development, interpretation, and manuscript writing. These roles are reported in the Methods and Authors’ contributions sections. |
| Interviewer/facilitator | Not applicable | No interviews or focus groups were conducted. |
| Credentials | Partially applicable | Author affiliation is provided on the title page. |
| Occupation | Partially applicable | Author affiliation in physical education is provided on the title page. |
| Gender | Not applicable | No interviewer-participant relationship was involved. |
| Experience and training | Partially applicable | The analytical approach was based on thematic analysis and public-document review. Specific personal training details were not central to the design. |
| Relationship established | Not applicable | No participants were recruited or contacted. |
| Participant knowledge of interviewer | Not applicable | No participants were involved. |
| Interviewer characteristics | Not applicable | No interview process was used. |
| Domain 2: Study design | Applicable / partially applicable | The study design, theoretical orientation, data sources, search strategy, inclusion and exclusion criteria, screening process, and source quality assessment are reported in the Methods section. |
| Methodological orientation and theory | Applicable | The study used retrospective qualitative thematic analysis informed by Braun and Clarke’s framework and a socio-ecological/systems-oriented perspective. |
| Participant selection | Not applicable | The study did not involve human participant recruitment. Case selection criteria are reported instead. |
| Sampling | Partially applicable | The study used a purposive public-case sample based on predefined inclusion and exclusion criteria. It was not intended to be statistically representative. |
| Method of approach | Not applicable | No participants were approached. |
| Sample size | Partially applicable | The main analytical sample included 21 publicly reported fatal cases. This number refers to cases, not participants recruited for qualitative interviews. |
| Non-participation | Not applicable | There was no participant recruitment or refusal. |
| Setting of data collection | Partially applicable | Data came from publicly available online materials, including university notices, official statements, and news reports. |
| Presence of non-participants | Not applicable | No interview or focus-group setting was involved. |
| Description of sample | Applicable | The 21 included cases are summarized in Table 2. Boundary cases and supplementary successful rescue cases are summarized in Tables 1 and 3. |
| Interview guide | Not applicable | No interviews were conducted. Instead, a structured data extraction framework was used. |
| Repeat interviews | Not applicable | No interviews were conducted. |
| Audio/visual recording | Not applicable | No interviews or focus groups were recorded. |
| Field notes | Partially applicable | The study used case files, extraction fields, coding notes, and an audit trail rather than field notes. |
| Duration | Not applicable | No interviews or focus groups were conducted. |
| Data saturation | Not applicable / partially applicable | Saturation in the interview-based sense was not applicable. The study aimed to identify recurrent patterns in a bounded public-case sample. |
| Transcripts returned | Not applicable | No transcripts were generated. |
| Domain 3: Analysis and findings | Applicable / partially applicable | The thematic analysis procedure, coding framework, theme development, source quality assessment, and coding examples are reported in the Methods and supplementary files. |
| Number of data coders | Applicable | Five trained coders assisted with preliminary coding. The author integrated coding outputs and conducted the final thematic analysis. Formal inter-coder reliability statistics were not calculated. |
| Description of coding tree | Applicable | The coding structure is provided in Additional file 2. Coding examples are provided in Table 5 and Additional file 2. |
| Derivation of themes | Applicable | Themes were developed through repeated reading, preliminary open coding, coding comparison, category grouping, cross-case comparison, and theme refinement. |
| Software | Partially applicable | No specialized qualitative analysis software was used; coding was conducted using structured extraction and coding tables. |
| Participant checking | Not applicable | No participants were involved, and no interview transcripts were produced. |
| Quotations presented | Partially applicable | Direct quotations were not emphasized to avoid unnecessary identification and because the data consisted of public reports. Instead, coding examples and summarized public descriptions are provided. |
| Data and findings consistency | Applicable | Themes were reviewed against original public source materials, and uncertain information was coded as “not reported” or “unclear”. |
| Clarity of major themes | Applicable | Six major themes are clearly reported in the Results section. |
| Clarity of minor themes | Partially applicable | Intermediate categories and codes are reported in Additional file 2. |

# 3. Summary of how COREQ was operationalized

In this study, COREQ was operationalized in a selective and design-appropriate manner. Items related to interviews, focus groups, participant recruitment, interviewer-participant relationships, audio-recording, transcription, and participant checking were not applicable because the study was based exclusively on publicly available documents.

However, the transparency-oriented principles of COREQ were addressed through the following procedures:

• The research design, theoretical orientation, data sources, search strategy, case screening process, and inclusion/exclusion criteria were explicitly reported.

• The public-case sample was described in detail, including fatal cases, boundary excluded cases, and supplementary successful rescue cases.

• Source quality was assessed according to source authority, proximity to the event, information completeness, cross-source consistency, and transparency of event description.

• The thematic analysis procedure was described step by step, including repeated reading, preliminary open coding, coding comparison, category development, cross-case comparison, theme refinement, and review against original source materials.

• A codebook, coding examples, and a theme–category–code linkage table were provided to improve analytic auditability.

• The methodological boundaries of the study were explicitly stated, including the inability to support causal inference, incidence estimation, or robust statistical association.

# 4. Statement on non-applicable COREQ items

Several COREQ items were not applicable to this study because the data were not generated through interviews or focus groups. Specifically, items concerning interviewer identity, relationship with participants, participant recruitment, interview setting, interview guide, repeat interviews, audio-recording, transcript return, and participant checking were not relevant to the present research design.

The absence of these procedures should not be interpreted as incomplete reporting, but as a reflection of the study’s public-document-based retrospective design.

This checklist is therefore not intended to claim full COREQ compliance, but to document which transparency principles were applicable and how they were addressed in a public-document-based qualitative design.
