# Supplementary material for: Contextual patterns in publicly reported non-traumatic sudden deaths among university students in physical education and campus sports settings in China: a retrospective thematic analysis
Source: BMC Public Health. 2026 Jun 8;26:2683. doi: 10.1186/s12889-026-28079-5 (PMC13615593; doi:10.1186/s12889-026-28079-5)
Supplement: Supplementary file 2 [file 12889_2026_28079_MOESM2_ESM.docx]

**Additional file 2. Codebook for thematic analysis**

Title: Codebook for the thematic analysis of publicly reported non-traumatic sudden death cases among university students in physical education and campus sports settings

# 1. Purpose of the codebook

This codebook was developed to improve the transparency and auditability of the thematic analysis. It documents how case-level information extracted from publicly available reports was organized into initial codes, intermediate categories, and higher-order themes.

The codebook was not designed to establish medical causality or statistical associations. Instead, it was used to identify recurrent contextual signals visible in public case materials, including reported health vulnerability, exercise-task demands, screening and monitoring information, psychosocial context, environmental conditions, and emergency response.

Because the study relied on publicly available reports rather than medical records, autopsy findings, or institutional investigation files, uncertain or missing information was coded as “not reported” or “unclear” rather than treated as evidence.

Five trained coders assisted with preliminary coding of the public case materials. The author developed the coding principles, compared and integrated the coding outputs, resolved ambiguous coding decisions with reference to the original public reports and codebook definitions, and conducted the final category construction, theme development, interpretation, and manuscript writing.

# 2. Coding principles

• Coding was based only on information available in public reports, university notices, institutional statements, education authority notices, or mainstream news reports.

• Public descriptions of cause of death were recorded as reported by the sources and were not interpreted as independent clinical determinations.

• Absence of information in a public report was not interpreted as absence of the phenomenon. For example, if no report mentioned AED use, the case was coded as “AED use not reported” rather than “AED not used”.

• When multiple sources described the same incident, more authoritative and information-rich sources were prioritized, including university notices, education authority notices, police or investigation statements, and established mainstream media reports.

• Codes were developed inductively from repeated reading of the case materials, but they were interpreted within a socio-ecological and systems-oriented perspective on campus health and safety.

• The final themes were interpreted as recurrent public-record-based contextual patterns or contextual signals rather than causal risk factors.

# 3. Codebook tables

Table A2.1. Codebook for thematic analysis

**Individual health vulnerability**

| **Code name** | **Operational definition** | **Inclusion rule** | **Exclusion rule** | **Example of coded information** |
| --- | --- | --- | --- | --- |
| Reported underlying disease | Public report mentions a known or suspected pre-existing disease, such as congenital heart disease, cardiac disease, myocarditis, or another serious health condition. | Code when disease history or underlying condition is explicitly mentioned by an official notice, investigation report, hospital description, family statement, or news report. | Do not infer disease when the report only says “sudden death” or “cardiac arrest” without further explanation. | A case report stated that the student had congenital heart disease and that death was related to the underlying condition. |
| Suspected cardiogenic event | Public source describes the event as cardiogenic sudden death, cardiac arrest, or sudden cardiac death. | Code when “cardiogenic sudden death”, “cardiac arrest”, or similar wording appears in public reports. | Do not code as confirmed cardiac disease unless medical diagnosis or investigation result is explicitly reported. | A report described the hospital diagnosis as “cardiogenic sudden death”. |
| Physical discomfort before collapse | Report mentions discomfort, abnormal symptoms, feeling unwell, collapse after symptoms, or poor physical condition before or during activity. | Code when discomfort or abnormal condition is described before collapse. | Do not infer symptoms when only sudden collapse is reported. | A student felt unwell during or after exercise before emergency rescue was initiated. |

**Mismatch between task demands and physical preparedness**

| **Code name** | **Operational definition** | **Inclusion rule** | **Exclusion rule** | **Example of coded information** |
| --- | --- | --- | --- | --- |
| Endurance running demand | Case occurred during endurance-related running, such as 800 m, 1000 m, group jogging, campus running, or fitness testing. | Code when the activity clearly involved running or endurance load. | Do not code general ball games unless running/endurance testing is specifically mentioned. | Collapse occurred during a 1000-meter physical fitness test. |
| Fitness-testing demand | Case occurred during institutionally organized physical fitness testing. | Code when the report identifies the setting as physical fitness test, 体测, or physical education fitness assessment. | Do not code informal exercise or sports competition as fitness testing. | The student collapsed during a university physical fitness test. |
| Competitive or selection pressure | Case occurred during a competition, sports meet, selection contest, or pre-competition training. | Code when the setting involved competition, selection, ranking, or preparation for competition. | Do not code ordinary physical education classes unless evaluative or competitive features are reported. | Collapse occurred during a sports meet selection event. |
| High-load or special training context | Case involved potentially high physiological load, such as intensive training, weight-cutting, pre-competition preparation, or prolonged exertion. | Code when the report describes training intensity, weight-cutting, or special preparation. | Do not infer high load solely from the name of the sport. | A student died during weight-cutting training before a boxing competition. |

**Limited visibility of health screening and classroom monitoring**

| **Code name** | **Operational definition** | **Inclusion rule** | **Exclusion rule** | **Example of coded information** |
| --- | --- | --- | --- | --- |
| Screening not reported | Public reports do not describe whether health screening, medical history review, exemption procedure, or risk stratification was conducted before the activity. | Code when no information on pre-exercise screening is available. | Do not interpret this as evidence that screening was absent. | The report described collapse during testing but did not mention prior screening or health review. |
| Warning signs not captured | Report suggests sudden collapse or abnormal condition, but does not provide evidence that early warning signs were identified or acted upon before the event. | Code when the report describes collapse without clear pre-collapse recognition or intervention. | Do not code if the report clearly states that symptoms were recognized and the student withdrew or received timely help before collapse. | Public materials stated that the student suddenly collapsed during running, with no details on prior warning signs. |
| Differentiated management unclear | Public reports do not clarify whether students with illness, poor condition, or known risk were exempted, adjusted, or monitored differently. | Code when risk stratification or task adjustment information is absent or unclear. | Do not infer poor management unless explicitly reported. | The report did not describe whether alternative testing or exemption options were available. |

**Incomplete public reporting of emergency preparedness and on-site rescue capacity**

| **Code name** | **Operational definition** | **Inclusion rule** | **Exclusion rule** | **Example of coded information** |
| --- | --- | --- | --- | --- |
| Emergency response reported | Case report mentions teacher rescue, student rescue, school clinic response, emergency call, ambulance arrival, hospital transfer, or CPR. | Code when any rescue or emergency response is described. | Do not code details that are not specifically reported. | The report stated that teachers performed rescue and called emergency services. |
| AED availability not reported | Public reports do not mention whether an AED was available or used. | Code when AED use or availability is absent from the report. | Do not interpret as AED absence unless explicitly stated. | The case described emergency rescue and hospital transfer but did not mention AED use. |
| Delayed or incomplete rescue chain | Report indicates delayed arrival, unsuccessful rescue, lack of vital signs when rescuers arrived, or incomplete information on immediate response. | Code when delay or incomplete rescue sequence is visible in the report. | Do not infer delay if timing is not reported. | The report stated that the student had no vital signs when rescue personnel arrived. |
| Successful rescue comparison | Supplementary case involved sudden cardiac arrest but survival after CPR, AED use, or coordinated emergency response. | Code only for supplementary successful rescue cases, not for the fatal main sample. | Do not include in the fatal-case analytical sample. | A university student was revived after AED defibrillation and CPR during a campus sports activity. |

**Evaluative contexts and possible barriers to symptom reporting**

| **Code name** | **Operational definition** | **Inclusion rule** | **Exclusion rule** | **Example of coded information** |
| --- | --- | --- | --- | --- |
| Evaluative exercise setting | Case occurred in a context involving grades, physical fitness testing, assessment, selection, competition, or institutional evaluation. | Code when the setting may have evaluative consequences. | Do not infer individual psychological pressure unless directly or contextually supported. | Collapse occurred during a 1000-meter fitness test. |
| Evaluative context with possible reporting barrier | The case occurred in an evaluative or competitive context in which reporting discomfort or stopping activity may have been difficult, although direct evidence of symptom concealment was not available. | Code only as contextual possibility when the setting is evaluative or competitive. | Do not state that the student concealed symptoms unless explicitly reported. | A student participated in a required fitness test before collapse. |
| Symptom concealment reported | Public material explicitly states or strongly indicates that the student concealed discomfort, continued activity despite illness, or did not report symptoms. | Code only when directly reported. | Do not infer concealment from sudden collapse alone. | A report states that the student continued activity despite feeling unwell. |

**Amplifying role of environmental conditions**

| **Code name** | **Operational definition** | **Inclusion rule** | **Exclusion rule** | **Example of coded information** |
| --- | --- | --- | --- | --- |
| Early-morning or special time context | Case occurred during morning exercise, pre-class activity, or another time context that may affect physiological readiness. | Code when the time context is reported. | Do not infer time if not reported. | Collapse occurred before morning exercise. |
| Temperature or seasonal exposure | Report mentions hot weather, cold weather, seasonal conditions, air quality, or other environmental stressors. | Code when environmental condition is explicitly mentioned. | Do not infer weather conditions based only on date or location unless verified. | The report mentioned exercise under hot or cold conditions. |
| Environmental information not reported | No public information is available about temperature, weather, air quality, or environmental context. | Code when environmental conditions are absent from the report. | Do not treat as normal environmental condition. | The case report did not mention weather or environmental conditions. |

**Cross-cutting source quality codes**

| **Code name** | **Operational definition** | **Inclusion rule** | **Exclusion rule** | **Example of coded information** |
| --- | --- | --- | --- | --- |
| Official source available | At least one university notice, education authority notice, police/investigation statement, or official institutional source was available. | Code when official source exists. | Do not code mainstream media as official source unless it reproduces an official statement. | The university issued a public statement about the incident. |
| Multiple-source consistency | Two or more sources report consistent basic information about date, institution, setting, activity, and outcome. | Code when core details are consistent across sources. | Do not code if reports conflict on core event details. | University notice and mainstream media report described the same setting and outcome. |
| Fragmentary information | Available reports are brief, incomplete, or missing key contextual variables. | Code when essential details such as setting, activity, or response are unclear. | Cases with insufficient essential information were excluded from the main sample. | Report mentions death but does not clearly identify the sports context. |

# 4. From codes to themes

Initial codes were compared across the 21 fatal cases and supplementary cases. Codes with conceptual similarity were grouped into intermediate categories. These categories were then reviewed and refined into six higher-order themes.

For example, the codes “reported underlying disease”, “suspected cardiogenic event”, and “physical discomfort before collapse” were grouped under the higher-order theme “individual health vulnerability”. The codes “endurance running demand”, “fitness-testing demand”, “competitive or selection pressure”, and “high-load or special training context” were grouped under the theme “mismatch between task demands and physical preparedness”.

The final themes were reviewed against the original case materials to ensure that they remained grounded in the public record. Theme names were deliberately framed as contextual patterns rather than causal risk factors.

# 5. Theme structure summary

The final thematic structure was as follows:

**Theme 1. Individual health vulnerability**

This theme captured publicly reported underlying disease, suspected cardiogenic events, abnormal health status, and physical discomfort before collapse.

**Theme 2. Mismatch between task demands and physical preparedness**

This theme captured running, endurance testing, competition, selection events, training, and other situations in which exercise-task demands may have exceeded students’ immediate physical preparedness or health condition.

**Theme 3. Limited visibility of health screening and classroom monitoring**

This theme captured the limited visibility of pre-exercise screening, risk stratification, differentiated management, warning-sign recognition, and monitoring processes in public reports.

**Theme 4. Incomplete public reporting of emergency preparedness and on-site rescue capacity**

This theme captured publicly reported emergency response, CPR, AED use or non-reporting, ambulance activation, hospital transfer, and rescue-chain completeness.

**Theme 5. Evaluative contexts and possible barriers to symptom reporting**

This theme captured evaluative, competitive, and institutional contexts in which students may have felt pressure to complete tasks or hesitate to report discomfort. This theme was interpreted cautiously because direct evidence of symptom concealment was often unavailable.

**Theme 6. Amplifying role of environmental conditions**

This theme captured environmental or temporal conditions, such as morning exercise, seasonal exposure, temperature, and other external conditions that may interact with individual and task-related risk.

# 6. Coding limitations

This codebook should be interpreted in light of the limitations of public-document-based analysis. The coding was based on publicly available reports rather than clinical records, autopsy reports, or confidential university investigation files. Therefore, the codes cannot verify medical cause of death, institutional responsibility, or the actual presence or absence of specific safety measures.

The code “not reported” indicates that information was absent from available public sources. It does not mean that the event, condition, or measure did not exist. Similarly, public descriptions such as “cardiogenic sudden death” or “cardiac arrest” were treated as reported descriptions rather than independent clinical diagnoses.

The purpose of the coding process was to support a transparent thematic synthesis of recurrent contextual patterns, not to establish causal pathways or epidemiological associations.
