# Supplementary material for: Contextual patterns in publicly reported non-traumatic sudden deaths among university students in physical education and campus sports settings in China: a retrospective thematic analysis
Source: BMC Public Health. 2026 Jun 8;26:2683. doi: 10.1186/s12889-026-28079-5 (PMC13615593; doi:10.1186/s12889-026-28079-5)
Supplement: Supplementary file 3 [file 12889_2026_28079_MOESM3_ESM.docx]

**Additional file 1. Case-level source inventory and source quality notes**

*Title: Case-level source inventory and source quality notes for publicly reported non-traumatic sudden death cases among university students in physical education and campus sports settings*

# 1. Purpose of this supplementary file

This supplementary file provides the case-level source inventory for the 21 publicly reported fatal cases included in the main analytical sample. It was prepared to improve the transparency, traceability, and auditability of the public-document-based case retrieval and screening process.

For each case, the file records the case ID, date, university, setting, source platform or source type, direct URL where available, additional source-checking status, key information available from public materials, and source limitations. The purpose of this file is not to verify medical causality or institutional responsibility, but to document the public sources on which the case-level extraction and thematic analysis were based.

Because this study relied on publicly available web-based materials rather than medical records, autopsy reports, or confidential institutional investigation files, public descriptions of cause of death were recorded as reported by the sources and were not treated as independent clinical determinations. Missing or unclear information was recorded as “not reported” or “unclear” rather than inferred.

# 2. Source identification and verification procedure

Potentially relevant cases were identified through public web searches, news reports, university notices, institutional statements, and other publicly available online materials. Searches were conducted primarily in Chinese because the events occurred in China and were mainly reported in Chinese-language sources.

For each potentially eligible incident, targeted follow-up searches were conducted using combinations of the institution name, date, activity type, and key event description. Multiple reports referring to the same incident were merged into one case file. Where several sources described the same case, priority was given to official university notices, education authority notices, police or investigation-related statements, and more information-rich mainstream media reports. Less authoritative sources were used only to supplement contextual information when they were consistent with higher-priority sources.

The following source quality dimensions were considered: source authority; proximity to the event; information completeness; cross-source consistency; and transparency of event description.

Cases based only on fragmentary, unverifiable, or non-sports-related information were excluded from the main analytical sample.

# 3. Source quality classification used in this file

Source type was classified as follows:

Official / institutional source: University notice, education authority notice, police or investigation-related statement, or institutional public statement.

Mainstream media source: Established national, provincial, or local news outlet that reported the event with identifiable case details.

Aggregated web source: Search-engine-indexed or reposted material, including web pages that preserved event information but were not themselves the original reporting institution.

Unclear or fragmentary source: Public information with limited traceability, insufficient contextual information, or unclear reporting origin. Cases relying only on such sources were not included in the main analytical sample.

Information completeness was classified descriptively rather than numerically. The purpose was to record whether key variables were available, including date, university, setting, activity, reported outcome, emergency response, health information, screening information, and source limitations.

# Table A1.1. Search strategy summary

| **Component** | **Search scope or search terms** |
| --- | --- |
| Time period | 1 January 2016 to 25 December 2025 |
| Geographic scope | Mainland China |
| Population | University students / college students |
| Main setting | Physical education classes, physical fitness tests, campus sports competitions, selection events, training sessions, extracurricular campus sports, and other clearly identifiable campus sports-related settings |
| Population and institution terms | 大学生; 高校学生; 高校; 大学; university student; college student |
| Physical education and sport terms | 体育课; 体测; 体质测试; 校园跑步; 篮球; 羽毛球; 运动会; 训练; 比赛; 选拔赛 |
| Event outcome terms | 猝死; 心脏骤停; 呼吸心跳骤停; 晕倒; 倒地; 抢救无效; 死亡 |
| Governance or incident terms | 通报; 情况说明; 学校回应; 安全事故; 调查 |
| Example search strings | 大学生 体育课 猝死; 高校 体测 猝死; 大学生 1000米 体测 猝死; 大学生 800米 猝死; 大学生 篮球 猝死; 高校 体育课 抢救无效; 大学生 运动会 猝死; 高校 学生 心脏骤停 死亡 |
| Final search update | 25 December 2025 |
| Search limitation | Public web searches are dynamic and duplicate-prone. This study does not claim systematic-review-style database counts, but provides case-level source inventory and screening logic. |

| **Case ID** | **Date** | **University** | **Setting / activity** | **Primary source or platform** | **Direct URL(s)** | **Source type** | **Key information available** | **Source limitations and quality notes** |
| --- | --- | --- | --- | --- | --- | --- | --- | --- |
| 1 | 2016-10-11 | Changzhou Institute of Light Industry Technology | Preparatory activity before physical education class | Sina News | Onlooker News  https://kknews.cc/other/mal8e8g.html | Mainstream media source | Date, university, PE-related warm-up setting, sudden death description, emergency call, on-site first aid, hospital transfer, unsuccessful rescue | Screening information not reported; AED availability not reported; medical cause based on public description only |
| 2 | 2016-11-04 | Chongqing University of Posts and Telecommunications | Physical education / physical fitness testing | Baidu Web / indexed public report | Gaokao Network  https://www.gaokao.com/e/20161107/58201b3c941ad.shtml | Aggregated web source / public report | Date, university, PE-related fitness testing context, collapse followed by death despite rescue, university response, hospital rescue, police involvement | Primary original source should be confirmed if available; screening process not reported; detailed emergency timeline not fully available |
| 3 | 2016-11-05 | Guangdong University of Technology | 800-meter race during campus / faculty sports meet | CYOL | Sina News https://news.sina.com.cn/c/2016-11-11/doc-ifxxsmuu5328217.shtml# | Mainstream media source | Date, university, sports meet setting, 800-meter race, hospital diagnosis described as cardiogenic sudden death, on-site rescue, emergency call, hospital rescue | Cause-of-death description is public-report-based; prior health history not fully reported; AED availability not reported |
| 4 | 2016-11-19 | Wuhan University | Physical fitness test | Baidu Web / indexed public report | Xinhua Net https://www.xinhuanet.com/politics/2016-11/28/c_1120001316.htm | Aggregated web source / public report | Date, university, physical fitness testing setting, sudden collapse during testing, on-site treatment, hospital transfer, death confirmed later | Sex not disclosed; medical cause not fully reported; screening, monitoring, and AED information not reported |
| 5 | 2017-02-17 | Guangxi University | Informal basketball on campus | The Paper / university statement reproduced | The Paper  https://www.thepaper.cn/newsDetail_forward_1622660 | Mainstream media source / institutional source | Date, university, extracurricular campus basketball setting, sudden death description, university notice and follow-up arrangements | Emergency-response details limited; screening not applicable or not reported; medical diagnosis not independently available |
| 6 | 2017-09-10 | Fuzhou Institute of Technology | Playing basketball on campus | Shaoxing News Online | TikTok News  https://www.toutiao.com/article/6464783039690965261/ | Local news source | Date, university, extracurricular basketball setting, initial sudden death description, campus clinic response, hospital transfer, unsuccessful rescue | Official source should be checked if available; detailed medical cause and screening information not reported; AED information not reported |
| 7 | 2018-01-04 | Zhejiang Institute of Mechanical and Electrical Engineering | Playing badminton | Baidu Web / indexed public report | ZJTV 1818 Golden Eye  https://baijiahao.baidu.com/s?id=1588746217065403015&wfr=spider&for=pc | Aggregated web source / public report | Date, university, extracurricular badminton setting, sudden death description, hospital transfer and unsuccessful rescue | Primary source traceability may be limited; screening information not reported; emergency-response process incomplete |
| 8 | 2018-04-26 | Tianjin University | Physical fitness test | China Online | WeChat Official Account  <https://mp.weixin.qq.com/s?__biz=MzA5MzE1Nzg0Mw==&mid=2651301389&idx>  =1&sn=5dc74c51d3b5a9f9fc9b76df3375f030&chksm=8b9126b1bce6afa720d0fb773ef7b9c8f5996cf668fc5097e7a31e0a41f49ab058cc1f30bc9e&scene=27 | Aggregated/web-based public source / online news source | Date, university, physical fitness testing setting, sudden collapse during testing, death despite rescue | Sex not disclosed; explicit medical cause not disclosed; screening, monitoring, AED, and detailed rescue timeline not reported |
| 9 | 2019-03-21 | Foshan University | Running on campus track | Sina News | Southern Metropolis Daily  <https://mp.weixin.qq.com/s?__biz=MTk1MjIwODAwMQ==&mid=2650796687&idx=>  1&sn=3af4fed614f56822dfc0d1fe28c64b4e&chksm=4468f161731f78770f0fcd00b238b5d78db879d9c1c0b8bc2a5ab627588e80d5cdc0e5c9cab3&scene=27 | Mainstream media source | Date, university, campus running setting, cardiac arrest description, on-site first aid, intensive hospital rescue, later brain death | Public description does not provide full clinical record; pre-existing disease and screening information not reported; AED use unclear |
| 10 | 2019-10-19 | Lushan College of Guangxi University of Science and Technology | 1000-meter physical fitness test | Baidu Web / indexed public report | Hndnews  https://www.hndnews.com/p/306335.html | Aggregated web source / indexed public report | Date, university, 1000-meter physical fitness test, on-site treatment, hospital rescue, police involvement | Medical cause not explicitly disclosed; original source should be verified if possible; screening and AED information not reported |
| 11 | 2019-10-27 | Xuzhou Medical University | 1000-meter running fitness test | CYOL | Baidu Web  https://baijiahao.baidu.com/s?id=1648905518487121378&wfr=spider&for=pc | Mainstream media source | Date, university, 1000-meter fitness test, hospital diagnosis described as sudden death, continuous chest compressions, hospital transfer, unsuccessful rescue | Cause-of-death description is public-report-based; prior health history not fully available; AED information not reported |
| 12 | 2019-11-09 | Inner Mongolia University of Finance and Economics | Running on west campus track | Baidu Web / indexed public report | Minnan News  http://www.mnw.cn/news/shehui/2218300.html | Aggregated web source / public report | Date, university, campus running setting, female student, sudden death description, university statement and campus safety follow-up | Primary official statement should be linked if available; screening and detailed emergency timeline not reported |
| 13 | 2021-04-29 | Baise University | Collapse near basketball court | Official website | Official Website of Baise University  https://www.bsuc.cn/info/1244/52272.htm | Official / institutional source | Date, university, basketball-court-related campus setting, family-cited hospital diagnosis of cardiogenic sudden death, hospital transfer, ICU admission, later death | Cause-of-death description partly based on family-cited hospital diagnosis; prior health and screening details not fully available |
| 14 | 2021-11-27 | Donghua University | Collapse after 1000-meter physical fitness test | Shaoxing News Online | Sina News  https://sh.sina.com.cn/news/z/nscpcs/?from=wap | Local news source / public report | Date, university, 1000-meter physical fitness test, sudden death / death despite rescue, on-site rescue, university suspended subsequent testing | Official university notice should be checked if available; AED use unclear; medical cause and screening information incomplete |
| 15 | 2022-03-24 | Shanxi University of Finance and Economics | Group jogging warm-up during PE class | Baidu Web / indexed public report | Baidu Web  https://baijiahao.baidu.com/s?id=1728514365857189840&wfr=spider&for=pc | Aggregated web source / indexed public report | Date, university, PE class context, group jogging warm-up, female student, teacher rescue, school hospital arrival, emergency transfer, working team established | Original university notice should be confirmed; medical cause, prior health history, AED availability, and screening information not fully reported |
| 16 | 2022-09-15 | Wuchang University of Technology | Playing basketball | China Online | CYOL  https://news.cyol.com/gb/articles/2022-09/26/content_zN4GbSYlq.html | Mainstream / online news source | Date, university, extracurricular basketball setting, hospital diagnosis described as respiratory and cardiac arrest, transfer for emergency treatment, unsuccessful rescue | Screening information not applicable or not reported; AED use and immediate rescue details incomplete; public diagnosis not independently verified |
| 17 | 2023-05-15 | A university in Nanchang | Sudden collapse while running during PE-related activity | Sina News | Baidu Web  https://baijiahao.baidu.com/s?id=1766695478358834427&wfr=spider&for=pc | Aggregated web source / indexed public report | Date, Nanchang university setting, running activity, preliminary diagnosis described as cardiogenic sudden death, emergency rescue unsuccessful, university response | University name not fully disclosed in table; preliminary diagnosis only; source should be triangulated with official response if available |
| 18 | 2023-09-03 | Guiyang Institute of Information Technology | Playing basketball | Baidu Web / indexed public report | official website  https://www.gyiist.edu.cn/gyit/article.jsp?id=25839&itemId=629 | Official / institutional source | Date, university, extracurricular basketball setting, preliminary sudden death description, possible cardiac cause, emergency rescue by 120 unsuccessful | Preliminary cause only; primary official or hospital source should be checked if available; screening and AED information not reported |
| 19 | 2024-04-12 | Baicheng Medical College | Sudden collapse before morning exercise | CYOL | Shaoxing News Online  https://www.shaoxing.com.cn/p/3186839.html | Mainstream media source / investigation-related report | Date, university, pre-class / morning exercise setting, female student, underlying congenital heart disease reported, death related to underlying disease, on-site first aid, emergency call, hospital transfer, personnel accountability mentioned | Stronger source detail than many cases due to reported underlying disease and accountability information; however, complete medical records and full institutional investigation file were not available |
| 20 | 2024-06-09 | Shenyang Normal University | Weight-cutting training before boxing competition | Baidu Web / indexed public report | Baidu Web  https://baijiahao.baidu.com/s?id=1801980980627736687&wfr=spider&for=pc | Aggregated web source / public report | Date, university, pre-competition training context, weight-cutting training before boxing competition, preliminary sudden death description, emergency personnel attempted rescue for about half an hour | Event involved collapse in dormitory after training context; sports-related boundary should be clearly justified; diagnosis preliminary; screening and supervision details incomplete |
| 21 | 2025-04-02 | Henan Mechanical and Electrical Vocational College | Running event in sports meet selection contest | Online news source / official statement reproduced | China Online  https://news.china.com/socialgd/10000169/20250408/48186382.html | Official / institutional source | Date, university, sports meet selection contest, running event, cardiogenic sudden death description, lost consciousness during race, no vital signs when rescue personnel arrived | Official/public information available, but medical diagnosis still based on public description; screening and AED availability not reported; detailed emergency timeline partly limited |

# Table A1.2. Case-level source inventory and source quality notes for the 21 included fatal cases

# 4. Notes on excluded boundary cases

Boundary cases were recorded separately to clarify the inclusion and exclusion logic. These cases were relevant to student mortality or campus safety but were excluded from the main analytical sample because they involved drowning, dormitory sudden death, campus facility accidents, unclear sports context, or insufficient contextual information to support thematic coding.

These boundary cases are summarized in Table 1 of the main manuscript and were used only to clarify the study boundary. They were not included in the thematic analysis of the 21 fatal cases.

# 5. Notes on successful rescue comparison cases

Successful rescue cases were collected as supplementary contextual materials related to emergency preparedness, AED deployment, CPR implementation, and campus rescue-chain organization. These cases were not included in the main fatal-case analytical sample because the outcome was survival rather than death.

They were used only to support prevention-oriented discussion about emergency response capacity and were summarized in Table 3 of the main manuscript.

# 6. General source limitations

First, public web searches are dynamic. Search results may change over time, some reports may become unavailable, and duplicate reports may appear across platforms. Therefore, this study does not claim systematic-review-style database counts.

Second, the completeness of public reporting varied substantially across cases. Some cases included official statements or relatively detailed descriptions of emergency response, whereas others were based mainly on news reports or indexed web materials.

Third, public descriptions of cause of death were not equivalent to independent clinical adjudication. Terms such as “sudden death”, “cardiogenic sudden death”, “cardiac arrest”, or “respiratory and cardiac arrest” were recorded as public-report descriptions only.

Fourth, information not reported in public sources was not treated as absent. For example, if a report did not mention AED use, the case was coded as “AED availability or use not reported” rather than “AED not available” or “AED not used”.

Fifth, this file is intended to improve transparency and traceability of the public-document-based analysis. It does not establish incidence, causality, institutional responsibility, or generalizability to all university sports settings in China.

# 7. Recommended reference in the main manuscript

A case-level source inventory, including source platform, direct URL, source type, key information available, and source quality limitations, is provided in Additional file 1.

Although a case-level source inventory and source quality notes are provided in Additional file 1, the analysis remains limited by the completeness, accessibility, and public-reporting practices of the available sources.
